# Performance in complex life situations: effects of age, cognition, and walking speed in virtual versus real life environments

**DOI:** 10.1186/s12984-021-00830-6

**Published:** 2021-02-08

**Authors:** Michal Kafri, Patrice L. Weiss, Gabriel Zeilig, Moshe Bondi, Ilanit Baum-Cohen, Rachel Kizony

**Affiliations:** 1 Department of Physical Therapy, Faculty of Social Welfare & Health Sciences, University of Haifa, Haifa, Israel; 2 Department of Occupational Therapy, Faculty of Social Welfare & Health Sciences, University of Haifa, Haifa, Israel; 3 Department of Neurological Rehabilitation, Sheba Medical Center, Tel Hashomer, Israel; 4 Sackler Faculty of Medicine, Tel Aviv University, Tel Aviv, Israel; 5 Health Professional Faculty, Ono Academic College, Kiryat Ono, Israel; 6 “Steps” Center for Rehabilitation, Sheba Medical Center, Tel Hashomer, Israel; 7 Occupational Therapy, Sheba Medical Center, Tel Hashomer, Israel; 8 Center of Advanced Technologies in Rehabilitation, Sheba Medical Center, Tel Hashomer, Israel

**Keywords:** Virtual environment, Virtual reality, Virtual shopping, Gait, Older adults, Simulation

## Abstract

**Background:**

Virtual reality (VR) enables objective and accurate measurement of behavior in ecologically valid and safe environments, while controlling the delivery of stimuli and maintaining standardized measurement protocols. Despite this potential, studies that compare virtual and real-world performance of complex daily activities are scarce. This study aimed to compare cognitive strategies and gait characteristics of young and older healthy adults as they engaged in a complex task while navigating in a real shopping mall and a high-fidelity virtual replica of the mall.

**Methods:**

Seventeen older adults (mean (SD) age = 71.2 (5.6) years, 64% males) and 17 young adults (26.7 (3.7) years, 35% males) participated. In two separate sessions they performed the Multiple Errands Test (MET) in a real-world mall or the Virtual MET (VMET) in the virtual environment. The real-world environment was a small shopping area and the virtual environment was created within the CAREN™ (Computer Assisted Rehabilitation Environment) Integrated Reality System. The performance of the task was assessed using motor and physiological measures (gait parameters and heart rate), MET or VMET time and score, and navigation efficiency (cognitive performance and strategy). Between (age groups) and within (environment) differences were analyzed with ANOVA repeated measures.

**Results:**

There were no significant age effects for any of the gait parameters but there were significant environment effects such that both age groups walked faster (F_(1,32)_ = 154.96, p < 0.0001) with higher step lengths (F_(1,32)_ = 86.36, p < 0.0001), had lower spatial and temporal gait variability (F_(1,32)_ = 95.71–36.06, p < 0.0001) and lower heart rate (F_(1,32)_ = 13.40, p < 0.01) in the real-world. There were significant age effects for MET/VMET scores (F_(1,32)_ = 19.77, p < 0.0001) and total time (F_(1,32)_ = 11.74, p < 0.05) indicating better performance of the younger group, and a significant environment effect for navigation efficiency (F_(1,32)_ = 7.6, p < 0.01) that was more efficient in the virtual environment.

**Conclusions:**

This comprehensive, ecological approach in the measurement of performance during tasks reminiscent of complex life situations showed the strengths of using virtual environments in assessing cognitive aspects and limitations of assessing motor aspects of performance. Difficulties by older adults were apparent mainly in the cognitive aspects indicating a need to evaluate them during complex task performance.

## Background

By 2035, twenty percent of the population will be 65 years or older [1]. This group strives for “successful aging” by aiming to maintain an active life style [2]. Models of successful aging recognize three main components including the prevention of diseases, good cognitive and physical functioning and engagement with life i.e. participation in daily activities [3, 4].

Older adults report that maintaining their ability to perform instrumental activities of daily living (IADL) such as driving and shopping are highly meaningful [5]. Eriksson et al. reported that IADL activities such as shopping in a store or for groceries were identified as important activities performed by more than 50% of older adults across Asian and Western countries [6]. The assessment of such complex tasks is challenging for many reasons including safety, accessibility, weather conditions and liability. As predicted by the models and supported by the empirical findings, physical and cognitive functions play a crucial role in aging successfully [7, 8]. Participation in daily activities, specifically those with high complexity such as shopping or driving are thus of great importance [9]. Participation restrictions in older adults has been associated with age-related physiological, cognitive and sensory-motor changes [10].

Evaluation of cognitive and motor functions in routine clinical procedures is based on a wide array of standardized clinical tests. However, such testing has limited ecological validity (i.e., minimal resemblance to everyday life demands) as it focuses on each motor or cognitive sub-domain in isolation, with little regard for ‘real-life’ demands and responses. Contrary to this ‘isolated’ approach, dual-task paradigms that assess concurrent motor-cognitive tasks such as texting [11] or calculating [12] while walking more closely replicate real-life activities, although such paradigms have been criticized for insufficient ecological validity [13–15] since they are often conducted in laboratory settings or entail non-functional tasks. We contend that a more valid evaluation of motor (specifically gait) and cognitive functions should be performed within the context of tasks that are relevant to older adult’s ‘real-life’ interests and activities, and should be documented via simultaneous recordings of both cognitive and motor performance. To date, neither of these recommendations has been sufficiently explored in the literature.

Assessment of an older person’s performance within simulations of daily life settings by means of laboratory-based virtual reality (VR) may overcome many of the above-mentioned barriers. VR enables objective and accurate measurement of behavior in ecologically valid and safe environments, while controlling the delivery of stimuli and maintaining standardized measurement protocols [15–17]. Despite this potential, studies that compared virtual and real-world performance of complex daily activities are scarce. Rand and colleagues [18], for example, examined the relationship between performance in the real world (via the Multiple Errands Test (MET) [19]) versus performance in a virtual shopping mall with an adapted version of the MET; the Virtual Multiple Errands Test (VMET) [18]. Both the MET and VMET assess executive functions during the performance of a complex shopping task. High, significant correlations were found between the performances of these two tasks, thus establishing the ecological validity of the VMET as a tool to assess executive functions. Nir-Hadad et al. [20] showed similar results when comparing a virtual shopping task to performance of the same task in the real environment. However, these studies focused on the cognitive aspect of performance and not gait or any other motor aspect.

In the study presented in this paper, we carried out a comprehensive, ecological approach that incorporated motor, cognitive and physiological aspects in the measurement of older adult’s performance during tasks reminiscent of complex life situations. The findings may lead to the development of ecologically valid assessments and interventions during in-situ daily life tasks [21] to maintain older adults’ participation in complex tasks in the community. The specific objectives were to: (1) compare performance (cognitive strategy and gait characteristics) of a complex task while navigating in a real shopping mall (MET) and a high-fidelity virtual replica of the mall (VMET); (2) to compare MET and VMET performance of young and older healthy adults; and (3) to examine within-group correlations between executive functions and performance scores in the MET and VMET. This work will lead to validation of the VMET relative to the MET.

## Method

### Study participants

Seventeen young and 17 older healthy adult participants were recruited via convenience sampling through advertisements published in relevant websites. Participants were included in the study if they were able to walk alone out-of-doors for at least 50 m without resting, and to shop for basic groceries. In addition, criteria for the elderly group were a score of 24 or above on the Mini Mental State Examination (MMSE) [22] and independence in basic activities of daily living (BADL). Any potential participant who had acute orthopedic disease, neurological deficits (e.g. acquired brain injury) and any medical illness that would prevent them from engaging in low effort exercise as confirmed by their family physician was excluded. An additional exclusion criterion for the older group was the presence of depression (a score below 5 on the Geriatric Depression scale (GDS)) [23].

### Instruments

*10-m Walk Test* (10-MWT) [24], to determine the overground comfortable walking speed.

*Trail Making Test* (TMT) [25] to assess visual-motor scanning and executive functions including divided attention and cognitive flexibility.

### Real world and virtual environments used for the measurements of a complex task

The real-world experiment was conducted at the Sheba Medical Center shopping mall during opening hours when it is usually crowded. This mall is a small, one-story shopping center that includes 15 clothing, jewelry, handbag and linen stores as well as a lottery ticket stand, fast-food restaurants, a coffee shop and a bank. The VR experiment was conducted at the Sheba Medical Center’s Center for Advanced Technologies and described in [26]. A virtual shopping mall was created within the CAREN™ (Computer Assisted Rehabilitation Environment) Integrated Reality System to simulate the real-world mall. The VE was a replica of the real mall in terms of store layout, scale and appearance.

The participant viewed the virtual mall projected onto a flat 52″ wall-mounted screen while walking on an interactive, self-paced instrumented treadmill (VGait; Motek Medical B.V.). The dimensions of the treadmill are 1.2 × 2 m. The participants were strapped to a trunk safety harness to safeguard against falls. The screen height (from the treadmill surface to the middle of the screen) was 168 cm and the distance between the screen and the participants was 167 cm. The monitor’s field of view was 36.4°. A digital clock showing the actual time appeared in the top, right hand corner of the screen. The participant navigated right, left, and backwards in the virtual mall using a joystick that was affixed to the right treadmill handrail. Participants were able to view their “virtual” hands on the monitor, using hand gestures to interact with virtual objects (e.g., “reaching” to “purchase” a newspaper located in one of the stores) in order to complete the required VMET tasks. Five passive markers were placed on anatomical landmarks including four on the pelvis (left and right anterior and posterior superior iliac spine) and one on the hand). These were detected by 12 VICON infra-red cameras (http://www.vicon.com). The pelvic markers were used to control the speed of the self-paced treadmill whereas the hand marker was used to control reaching of items within the VE. A non-functional simulation that was similar in layout but with no active stores was created as well.

### Tasks performed within the study environments

In the real-world environment, the MET– Simplified Version [19] was performed. The participant is asked to carry out three types of tasks (divided into sub-tasks) in a shopping center: purchase six items, obtain four items of information, and meet the examiner at a preset location and time, all while abiding by specific rules. The examiner observes the participant to provide a score based on strategies and mistakes (detailed below). This assessment has high interrater reliability (0.81–1.00). Discriminant validity was established between people with traumatic brain injury (TBI) and healthy people and between people with TBI and people with schizophrenia [19, 27]. In addition, ecological validity in relation to daily functioning has been established [28].

In the current study, participants were instructed how to perform the task including the specific rules, and then given a detailed list of all task, components, a small pouch with money and a pencil to record information as necessary. A version of the MET adapted for use in a virtual environment, the VMET [18] was performed in the virtual environment. It incorporated the same number and type of tasks as for the MET with one exception; whereas the participants were instructed to meet the examiner in the MET at a preset time and location, in the VMET the participants were asked to tell the examiner how many products they purchased at a preset time. The list of VMET tasks to be performed was written on a poster in large black block letters that was affixed to the bottom edge of the monitor.

### Outcome measures

Cognitive, motor and physiologic variables of MET/VMET task performance were recorded. Cognitive evaluation of task performance was based on the scoring of the MET/VMET, which included the measures of performance time as described by Morisson et al. [29] and of the number of mistakes observed by the examiner [30]. Errors include errors in task completion (categorized as non-performance or partial-performance), errors of partial failure to perform the task (i.e. self-correction of errors in task performance), errors of inefficient task performance (i.e. in searching for a product in inappropriate locations), errors in failing to abide by the rules (i.e. entering the same store twice), and errors in strategy use. The total error score ranges from 0 to 133. A low score denotes adequate performance whereas a high score denotes deficient performance. In addition, a navigation efficiency score that reflects the strategy the participants used to plan their task, was calculated; each task’s location that was reached directly from the location of previous nearest task was scored as “1”. The total score was the sum of all navigation efficient tasks divided by the number of total tasks and multiplied by 100. Higher scores denote greater efficiency. Total number of strides was used as an adjunct measure to the efficiency score in order to illuminate the gait aspect for this score; higher number of strides reflect less efficient planning.

*Gait variables* speed, stride time and length and their coefficient of variances, which represent motor performance and are considered as markers for functional decline due to aging in single [31] or dual tasks [32]. These were measured continuously during both tasks using the Mobility lab System (http://www.apdm.com/gait-and-posture/Mobility-Lab/). Three wireless OPAL™ movement monitors were affixed to the participant’s legs and waist (Mobility Lab, APDM Inc, Portland, Oregon).

Heart rate (HR) was continuously measured using a Polar HR monitor (Polar Electro, Kempele, Finland). Rate of perceived exertion (RPE) was recorded at the end of either MET or VMET performance using Borg’s RPE scale of 6–20 [33].

### Procedure

Participants who met the inclusion criteria signed an informed consent that was approved by the Helsinki committee of the Sheba Medical Center. They participated in two sessions, each lasting approximately 2 h. During the first session, the MMSE and GDS were administered to the elderly participants. Those participants who met the inclusion criteria completed the Personal Details Questionnaire. Next, both groups were administered the 10-MWT and the TMT. Then, each participant performed the MET assessment in the real-world (RW) mall or the VMET assessment in the virtual environment (VE). During the second session, the participants performed the assessment they had not performed during the first session. The sequence of assessments was alternated to minimize sequencing bias. Prior to the performance of the MET or VMET, participants were instrumented with the measurement systems including the OPAL sensors and the HR monitor. In addition, prior to the performance of the VMET participants were given 5–10 min to practice walking on the treadmill at their own comfortable speed with the goal of reaching a speed of at least 1 m/s and to learn to voluntary change gait speed and stop walking. In addition, they were given 5–10 min of practice time in the VE so that they would become familiar with the simulated shopping mall and how to interact within it (e.g., select items to buy) while walking on the treadmill.

### Data analysis

In both environments, gait variables (velocity, stride length, stride time) and their coefficients of variation were calculated from the APDM sensors. Analysis was performed for sequences with more than 5 strides. The gait parameters were analyzed using a customized code written in MATLAB R2011b (The MathWorks, Natick, MA). To compare performances between environments and between age groups, analysis of variance (ANOVA) repeated measures mixed design with one within-subject variable (environment) and one between-subject variable (group) were performed for each of the gait and cognitive outcome measures. Pearson correlation testing was used to analyze relationships between MET and VMET performance, gait and executive functions as measured by TMT. Due to technical difficulties, the VMET scores for two older adults and one young adult were not saved. In addition, the TMT A & B scores for one participant from the older group were deemed to be an outlier and therefore correlations with the TMT were performed without results from this participant.

## Results

Seventeen older adults (mean (standard deviation (SD) age = 71.2 (5.6) years, 64% males, mean (SD) years of education: 14.4 (2.5)) and 17 young adults (mean (SD) age: 26.7 (3.7) years, 35% males, mean years of education: 15.5 (2.4)) participated in the study. No significant between-group differences were found for gender and education. The mean (SD) MMSE score of the elderly participants was 29.1 (0.9), close to the maximal score of 30, and thus indicative of intact cognitive abilities. Their mean (SD) GDS assessment score was 0.88 (0.9) indicating an absence of depression.

### Gait and physical effort

Mean (SD) overground baseline walking speed as measured by the 10-MWT did not differ significantly between groups (young: 1.43 (0.04) m/s; older adults: 1.32 (0.06) m/s).

Table 1 summarizes the gait and aerobic variables according to the two age groups and two environments (RW and VE). There were no significant age effects for any of the gait characteristics but there were significant environment effects such that both age groups walked faster, took longer steps and had lower gait variability in the RW. The effect sizes of the environment for gait characteristics, as measured by partial eta square, were high, ranging from 0.53 to 0.83. There was a significant age and environment interaction effect for stride time; however, post-hoc analysis of age groups between the VE and RW showed shorter stride times for both groups in the RW (young t(16) = 9.17, p < 0.001 and older t(16) = 6.46, p < 0.001).

**Table 1:** Gait and aerobic variables according to groups and environments

	Young (n = 17) (Mean ± SD)		Older (n = 17) (Mean ± SD)		F environment (1,32) ES	F age (1,32) ES	F interaction (1,32) ES
	VE	RW	VE	RW			
Gait speed (m/s)	0.50 ± 0.14	1.23 ± 0.29	0.48 ± 0.15	1.18 ± 0.34	154.96*** 0.83	NS	NS
Stride length (m)	0.74 ± 0.20	1.33 ± 0.31	0.67 ± 0.20	1.32 ± 0.35	86.36*** 0.73	NS	NS
Stride time (s)	1.51 ± 0.18	1.09 ± 0.07	1.41 ± 0.17	1.14 ± 0.08	123.54*** 0.79	NS	5.46* 0.15
Stride length variability (CoV)	0.23 ± 0.09	0.09 ± 0.044	0.25 ± 0.06	0.091 ± 0.05	95.71*** 0.75	NS	NS
Stride time variability (CoV)	0.09 ± 0.02	0.07 ± 0.01	0.10 ± 0.03	0.07 ± 0.02	36.06*** 0.53	NS	NS
					DF_(1,28)_		
Heart rate (bpm)	87.88 ± 13.91	96.36 ± 13.60	80.53 ± 12.92	85.64 ± 12.50	13.40** 0.32	4.1* 0.13	NS
					DF_(1,31)_		
Borg (score)	10.41 ± 2.40	9.71 ± 2.081	10 ± 2.50	7.88 ± 1.54	13.36 0.30	NS	NS

BPM, beat per minute; CoV, coefficient of variance; DF, degrees of freedom; ES, effect size (partial eta square); RW, real world; SD, standard deviation; VE, virtual environment

*p ≤ 0.05 **p ≤ 0.01 ***p ≤ 0.0001

A significant age effect was found for HR but not for perceived effort (i.e. the Borg Scale); older participants exerted significantly less aerobic effort as measured by their HR but did not differ from the young adult participants in their rating of perceived effort. Significant environment effects were found for HR and perceived effort; HR was significantly higher but perceived effort was significantly lower in the RW. The effect sizes for these measures was lower than for the gait characteristics (partial eta square of about 0.32). There were no significant age and environment interaction effects for HR and perceived effort.

### Cognitive domain

Table 2 summarizes the cognitive variables according to the two age groups and two environments (RW and VE). Significant age effects were found for MET and VMET scores indicating better performance of the younger group, and for the number of strides that was larger in the older group. The effect size of age as measured by partial eta square ranged from 0.19 to 0.41. A significant environment effect was found only for the efficiency score that was higher in the VMET, although the time to complete the MET (longer) than the VMET showed an effect approaching significance. There were no age and environment interaction effects for any of the variables.

**Table 2:** Cognitive domain results

	Young (n = 17) (Mean ± SD)		Older (n = 17) (Mean ± SD)		F environment (1,29) ES		F age (1,29) ES		F interaction (1,29) ES
	VE	RW	VE	RW					
MET/VMET Score	2.06 ± 1.34	1.94 ± 1.34	5.07 ± 2.74	4.20 ± 2.01	NS		19.77*** 0.41		NS
MET/VMET total time (min)	9.63 ± 3.67	11.44 ± 2.33	14.25 ± 4.62	15.09 ± 4.47	NS		11.74* 0.29		NS
Navigation efficiency (%)	84.31 ± 14.99	61.03 ± 17.05	68.89 ± 18.76	59.17 ± 17.97	16.21*** 0.36		NS		NS
Total number of strides	76.06 ± 37.14	75.94 ± 53.24	106.78 ± 48.89	111.24 ± 47.85	NS		7.60** 0.19		NS

Due to technical problems two older adults and one young adult did not have their VMET scores. Degrees of freedom for the total number of strides are 1,32

ES, effect size (partial eta square); MET, Multiple Errands Test; NS, non-significant; RW, real world; SD, standard deviation; VE, virtual environment; VMET, virtual MET.

*p ≤ 0.05 **p ≤ 0.01 ***p ≤ 0.0001

### Correlations with TMTs

Within the older group, significant correlations between TMT A and RW gait speed (r =  − 0.51, p = 0.04) and TMT A and stride time (r = 0.56, p = 0.02). TMT B correlated significantly with MET total time (r =  − 0.53, p = 0.02), RW gait speed (r = 0.62, p = 0.01) and stride length coefficient of variance (r = 0.61, p = 0.01).

## Discussion

We carried out a comprehensive, ecological approach to the measurement of performance in complex life situations, comparing between performance in real versus virtual environments as well as between older and younger adults. The effect of environment was more pronounced in the motor aspects of performance whereas the effect of age was more pronounced in the cognitive aspects. Walking while shopping in a virtual mall resulted in lower gait speeds, shorter step lengths, longer stride times and higher spatio-temporal variability compared to the same task in a real mall. These outcomes were accompanied by a lower HR in the VE indicating an overall lower level of physical exertion.

Several factors may account for these observations. First, there are known differences between walking overground and walking on a treadmill, even self-paced models [14]; this is likely due to disparities in sensory feedback such as contact with the ground and optic flow [34]. Our findings are in accordance with studies that showed differences in spatio-temporal parameters and muscle activity between overground and treadmill walking, which had previously been examined primarily with fixed-paced treadmills [35, 36]. However, there are inconsistencies in the literature with some studies showing that there are no differences between treadmill versus overground walking [37].

The unique features of the current study may shed light regarding the treadmill versus overground walking differences found. In the current study, walking on the treadmill, i.e., the VE condition, was accompanied by sensory feedback such as visualization of the virtual stores and items to purchase. Although great effort was expended to achieve realistic graphical representation in the VE, visual feedback differed between the RW and VE, regardless of whether the participant walked overground or on a treadmill. The difference was intensified by the novelty of virtual shopping, especially for individuals who are not familiar with VR technology. For example, Sloot et al. [38] demonstrated that the addition of VR to a self-paced treadmill walking led to participants employing greater caution relative to overground walking that was reflected, for example, by reduced step length. Kizony et al. [14] found that in comparison to an overground paradigm, walking on a self-paced treadmill with VR was slower and had higher gait variability for both healthy and post-stroke adults. In addition, it is possible that in the current study, the adaptation of the participants to the treadmill walking was not complete. Meyer et al. [34] argue that 6 min of treadmill walking is the minimum duration required to achieve an adaptation plateau. We note that in the current study, participants practiced treadmill walking for 5 − 10 min to avoid this potentially confounding effect.

An increased cognitive load in the VE may also contribute to differences with the RW. Performance in the VE required walking while manipulating a joystick to navigate in the environment. It also required controlling the speed of the self-paced treadmill in order to gradually decrease the belt’s motion, as stopping a self-paced treadmill is not instantaneous. These tasks are not natural for walking, requiring attention and planning, and thus are similar to performing a secondary task while walking. As indicated by Sloot et al. [39], increases in cognitive load that occur in dual-task paradigms are associated with a reduction in walking velocity, cadence and stride length as well as an increase in stride time.

The findings of the current study further demonstrated the challenge of achieving physical effort during VE actions that is comparable to equivalent tasks in the RW. This may be attributed to the slower gait speed and is in accordance with studies that have shown lower energy expenditure while exercising in VE compared to RW. [40]

The absence of age effects on gait parameters in both environments may reflect the characteristics of the tasks (MET and VMET) that require only mild physical effort (e.g. participants can pause at will) while walking on flat surfaces with no perturbations. In line with our findings, Janouch et al. [15] showed that age did not affect walking speed when performing a virtual street crossing task.

In the cognitive domain, the results showed a different pattern with greater effects of age rather than environment. These results indicate that the cognitive demands of the task in both environments were sensitive to age-related differences associated with performing complex daily tasks. Indeed, the open-ended naturalistic task of the MET requires the use of various executive functions such as problem solving, inhibition, working memory and planning to be completed successfully and efficiently [41]. The need for adequate executive functions is reflected by the MET scores that were significantly higher in the older adults indicating inefficient task performance and strategy use. As reported in previous studies [18, 42], VMET scores behaved similarly to the MET with both requiring abilities that decrease due to the aging process [43, 44]. In keeping with these results, the longer time to complete the task by the older adults, which was not accompanied by slower gait speed compared to the young adults, suggests that the older adults also demonstrated slower information processing, a well-recognized, age-dependent process [45].

Navigation efficiency was measured using two complementary outcomes; sequencing of sub-tasks and number of strides as a proxy measure for distance traversed. The sequencing score was the only score in the cognitive domain that was affected by the environment, and not by age, with an advantage to the VE in both groups. In the current study, this score was based on efficient sequencing of the sub-tasks that involve pre-planning according to store location in the mall and was based on the actual execution of each sub-task of the MET or VMET. It appears that, in the VE, the user’s field of view enabled the “capture” of more store locations simultaneously with no interference by, for example, people walking nearby and blocking the visual scene; it was thus it was easier to plan the sub-task’s sequencing.

In contrast to the score of sequencing, number of strides was affected by age but not by environment, despite the between-group similarity in stride length. Trajectories (walking distance) represent the ability of a person to efficiently plan and use cognitive strategies (such as organizing and prioritizing sub-tasks with a checklist) and have been shown to distinguish between healthy people and those who have sustained a stroke [20]. The current findings suggest that older adults wander about in the mall before executing each step of the task, thus taking longer to complete the MET or VMET. These contrasting findings reinforce the importance of using a comprehensive approach for measuring performance efficiency during complex daily activities. Specifically, our results on walking distance and number of strides highlight the increased sensitivity to age-related difficulties during complex task performance experienced by community-dwelling older adults. These measures are easier to document via tasks performed in VR.

To achieve ecological validity of testing, sufficient levels of biomechanical fidelity are necessary. Biomechanical (motor) fidelity is defined as movements that are made with similar temporal and spatial parameters, muscle activation patterns, and joint forces in both the VE and a RW [46]. An additional source of demonstrating fidelity can be obtained from surrogate measures of motor performance including energy expenditure and neural activity [47]. Overall, the differences in motor and physiological outcomes between the environments found in the current study suggest that the motor fidelity of the VE while performing the VMET was low. This has some implications when considering the use of this or similar platforms to treat motor aspects of gait and/or physical effort. The findings highlight the need for clinicians to combine treatment in RW and VE settings as well as to encourage patients to self-practice in the RW with the goal of facilitating transfer between VR and RW. This recommendation is supported by principles of rehabilitation that emphasize the need to explicitly practice in real environments in order to foster transfer of skills trained within a clinical setting [48].

Recent studies used head-mounted displays (HMDs) to enable overground walking in a VE thereby circumventing the need to ambulate via a treadmill. Such devices provide an avenue to design VR activities with potentially stronger motor fidelity. This approach, however, still lacks complete RW authenticity due to space limitations that force participants to ambulate in a round walkway [49]. Moreover, wearing an HMD is associated with some encumbrance, and leads to side effects such as dizziness in some users (although the newer, faster models appear to provide a more satisfactory user experience) [50]. Future developments of VR for rehabilitation should make use of technological solutions and advances to enhance motor fidelity, such as using advances in HMD that have higher refresh rates to minimize side-effects.

With regard to the cognitive domain, the results of the current study support the fidelity of using a VE to measure performance of older adults during complex daily activities. However, the lack of correlations between the VMET and TMT challenge this notion. TMT is used extensively in research due to its predictive validity of functional ability or risk (e.g. of falling) in older adults and clinical populations when engaged in activities in the real world [51]. Our results appear to point to the nature of performance within the VE that perhaps relates to differences in the relationship between cognitive and motor demands as opposed to their relationship in the RW.

Limitations of this study include its relatively small sample size although both between- and within-group significant differences were found. The healthy older adult participants were high functioning in their motor and cognitive abilities, and a clinical population was not tested. Some aspects of the VE including auditory stimuli, lighting and presence of crowd did not replicate the real world. The inclusion of short sequences of walking may have influenced the gait analysis results, specifically gait variability.

## Conclusions

The current study presented a comprehensive approach for measuring performance during a range of meaningful daily complex tasks in RW compared to VR. The fidelity of using VR for this purpose, especially when motor-cognitive tasks are evaluated, needs further exploration to ensure that simulation of the real word is sufficiently authentic in the VE. Our findings show that age-related changes may be probed by the cognitive aspect of performance of daily complex tasks such as shopping in a mall. Future studies should examine ways in which this comprehensive approach facilitates the concurrent evaluation of motor and cognitive aspects during daily activities in older adults and clinical populations while simultaneously manipulating the motor and cognitive demands of the task.

## Data Availability

The datasets generated during and/or analyzed during the current study are not publicly available due to policy restrictions that were dictated during the time the study was approved by the ethic committee, but are available from the corresponding author on reasonable request.

## Abbreviations

IADL: Instrumental activities of daily living
MET: Multiple Errands Test
VMET: Virtual Multiple Errands Test
MMSE: Mini Mental State Examination
GDS: Geriatric Depression Scale
10-MWT: 10 M Walk Test
CAREN: Computer Assisted Rehabilitation Environment
TBI: Traumatic brain injury
HR: Heart rate
RPE: Rate of perceived exertion
RW: Real-world
VE: Virtual environment
ANOVA: Analysis of variance
SD: Standard deviation
HMD: Head-mounted display
